# Antimicrobial Peptides: Identification of two Beta-Defensins in a Teleost Fish, the European Sea Bass (*Dicentrarchus labrax*)

**DOI:** 10.3390/ph14060566

**Published:** 2021-06-14

**Authors:** Carolina Barroso, Pedro Carvalho, José F. M. Gonçalves, Pedro N. S. Rodrigues, João V. Neves

**Affiliations:** 1 i3S—Instituto de Investigação e Inovação em Saúde, Universidade do Porto, 4200-135 Porto, Portugal; prodrigu@ibmc.up.pt (P.N.S.R.); jneves@ibmc.up.pt (J.V.N.); 2 Iron and Innate Immunity, IBMC—Instituto de Biologia Molecular e Celular, Universidade do Porto, 4200-135 Porto, Portugal; 3 Programa Doutoral em Biologia Molecular e Celular (MCbiology), ICBAS—Instituto de Ciências Biomédicas Abel Salazar, Universidade do Porto, 4050-313, Porto, Portugal; 4 ICBAS—Instituto de Ciências Biomédicas Abel Salazar, Universidade do Porto, 4050-313 Porto, Portugal; pecarvalho@icbas.up.pt (P.C.); jfmg@icbas.up.pt (J.F.M.G.); 5 CIIMAR—Centro Interdisciplinar de Investigação Marinha e Ambiental, Universidade do Porto, 4450-208 Porto, Portugal

**Keywords:** aquaculture, European sea bass (*Dicentrarchus labrax*), antimicrobial peptides, beta-defensins

## Abstract

Beta-defensins consist in a group of cysteine-rich antimicrobial peptides (AMPs), widely found throughout vertebrate species, including teleost fish, with antimicrobial and immunomodulatory activities. However, although the European sea bass (*Dicentrarchus labrax*) is one of the most commercially important farmed fish species in the Mediterranean area, the characterization of its beta-defensins and its potential applications are still missing. In this study, we characterized two members of the beta-defensin family in this species. Phylogenetic and synteny analysis places sea bass peptides in the beta-defensin subfamilies 1 and 2, sharing similar features with the other members, including the six cysteines and the tertiary structure, that consists in three antiparallel beta-sheets, with beta-defensin 1 presenting an extra alpha-helix at the N-terminal. Further studies are necessary to uncover the functions of sea bass beta-defensins, particularly their antimicrobial and immunomodulatory properties, in order to develop novel prophylactic or therapeutic compounds to be used in aquaculture production.

## 1. Introduction

Defensins are a group of small cysteine-rich and cationic antimicrobial peptides (AMPs) found throughout nature. These peptides are divided into three different types: alpha-, beta- and the cyclic theta-defensins, a classification based on the cysteine pairing to form intramolecular disulphide bonds [1]. However, in phylogenetically earlier vertebrates such as teleost fish, only one defensin type has been isolated. Fish defensins present six conserved cysteines linked in a particular pattern of Cys1–Cys5, Cys2–Cys4 and Cys3–Cys6 [2,3,4,5], resembling the beta-defensin family members found in birds, reptiles and mammals [6,7,8,9]. Thus, while alpha- and theta-defensins appear to be more restricted to mammals, with theta-defensins being found only in some non-human primates [10,11], beta-defensins are widely distributed in different vertebrate species.

Beta-defensins were first isolated in zebrafish (*Danio rerio*), tiger pufferfish (*Takifugu rubripes*) and spotted green pufferfish (*Tetraodon nigroviridis*) [2]. These AMPs were later found in several other fish species, with only one gene copy being characterized in Atlantic cod (*Gadus morhua*) [4] or Nile tilapia (*Oreochromis niloticus*) [5], to the seven defensins isolated in the Atlantic salmon (*Salmo salar*), distributed into five different subfamilies [12]. In zebrafish, three beta-defensins were isolated, with the beta-defensin 3 gene being found in a different chromosome, indicating that multiple defensin loci are present in this fish species [2]. Fish defensin genes are formed by three-exons/two-introns, differing from the mammalian counterparts, that are encoded by two exons [2,5,12,13]. The resulting prepeptide is composed by a signal peptide with 18 to 26 amino acids and a 39 to 45 mature peptide [2,3,4,12,14,15], the defensins isolated in olive flounder (*Paralichthys olivaceus*) being an exception. This fish species presents a particular group of beta-defensins, constituted by peptides with 67 to 77 amino acids, in which a 5 to 15 amino acid proregion is also present that is cleaved to produce the active protein, which shows anionic properties [16]. Nevertheless, fish beta-defensin mature peptides share the six conserved cysteines and have an overall cationic net charge, folding into three anti-parallel beta-strands stabilized by the disulfide bonds, although some peptides may also possess an extra alpha-helix at the N-terminus of the three beta-strand structure [2,4,5,17,18].

Fish beta-defensins have been shown to be involved in several functions, including antibacterial, antiviral and immunomodulatory activities [19]. A high basal expression of beta-defensin genes can be observed in different tissues, including the spleen, gills, kidney or skin [5,20], and their expression is altered after stimuli with pathogen-associated molecular patterns (PAMPs), or by infection with different pathogens [21,22,23]. In vitro, recombinant or synthetic defensins are able to inhibit the growth of Gram-positive and Gram-negative bacteria, as well as some viruses [24,25,26]. The immunomodulatory functions of vertebrate beta-defensins include enhanced phagocytic activity [4,27], chemotaxis of fish leukocytes [14,28] and human immature dendritic cells and T cells [29], and also modulation of immune-related genes [28].

The European sea bass (*Dicentrarchus labrax*) is one of the most commercially important and intensively farmed fish species, particularly in the Mediterranean area. These fish are often subjected to disease outbreaks, mainly of bacterial and viral origin [30], and the current prophylactic and therapeutic approaches to deal with these outbreaks are limited. As such, novel compounds are necessary, with AMPs being promising candidates [31]. However, a comprehensive study of the different AMPs in sea bass is still lacking, and, thus, in this study, we characterize two beta-defensin family members in sea bass. These peptides fall into two different types, showing high similarities with other Perciform beta-defensins in terms of amino acid sequences and structure. Further studies are necessary to understand the functions of these peptides, in order to develop novel potential drugs to prevent or treat diseases in sea bass aquaculture.

## 2. Results

### 2.1. Molecular Characterization of Sea Bass Beta-Defensins

**Figure 1:**
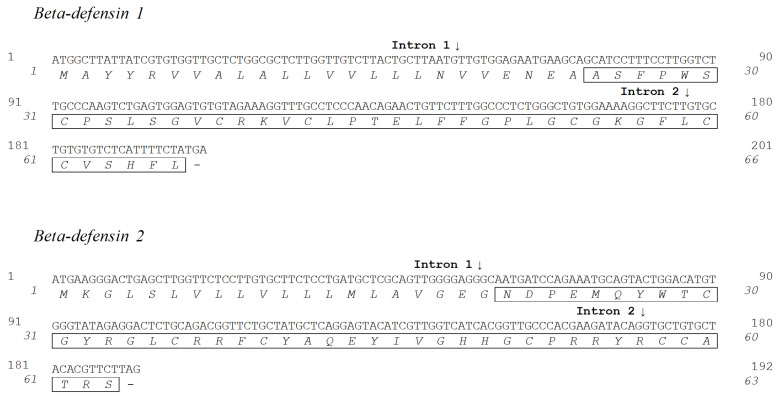
Sea bass beta-defensin coding DNA and amino acid sequences. Nucleotides are indicated in the upper row, and amino acids are indicated in italic in the lower row. Mature peptides are boxed, and intron positions are indicated by arrows.

Two different beta-defensin genes were obtained by PCR amplifications using intestine, kidney and gill cDNA (Figure 1). Potential cleavage sites were determined using SignalP-5.0 (http://www.cbs.dtu.dk/services/SignalP/, accessed on April 2021).

Beta-defensin1 coding DNA (deposited in GenBank under accession number MZ198753) consists of an open reading frame (ORF) of 201 bp and encodes a 66-aa prepeptide. A potential cleavage site for the signal peptide was predicted between Ala24 and Ala25 (NEA/AS). Thus, the beta-defensin1 prepeptide consists of a 24-aa signal peptide and a 42-aa mature peptide. The mature peptide has a predicted M.W. of 4493.4 Da, an isoelectric point of 8.13 and a net charge at pH 7 of 1.8.

Beta-defensin2 coding DNA (accession number MZ198754) consists of an ORF of 192 bp, and encodes a 63-aa prepeptide. The potential cleavage site for the signal peptide was predicted between Gly20 and Asn23 (GEG/ND). Thus, beta-defensin2 is formed by a 20-aa signal peptide and a 43-aa mature peptide. The mature peptide has a predicted M.W. of 5205.9 Da, an isoelectric point of 8.77 and a net charge at pH 7 of 3.9.

**Figure 2:**
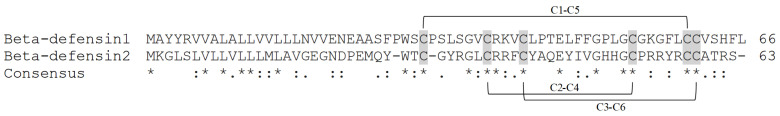
Alignment of sea bass beta-defensins. Cysteines are shaded gray, and the predicted disulphide bridges are also shown. Identical residues are denoted by (*), conserved substitutions by (:) and semi-conserved substitutions by (.).

Alignment between seabass beta-defensin peptides revealed several amino acid differences (identity score of 29.4%). However, both mature peptides retain the characteristic six cysteine residues at conserved positions. These six cysteines are predicted to form intramolecular disulphide bonds between Cys1–Cys5, Cys2–Cys4 and Cys3–Cys6 (Figure 2).

### 2.2. Molecular Modeling of Sea Bass Beta-Defensin Peptides

**Figure 3:**
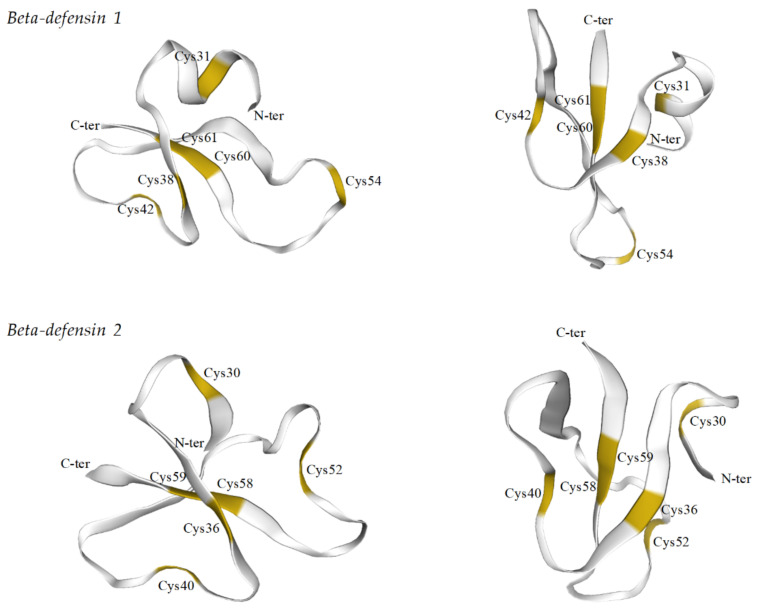
Three dimensional models of sea bass beta-defensins 1 and 2. Conserved cysteines are highlighted. Beta-defensin 1 shows an additional alpha-helix at the N-terminal. Models were predicted using as templates human beta-defensin 6 (2lwl.1.A) for beta-defensin 1, and human beta-defensin 4 (5ki9.1.A) and oyster big defensin (6qbk.1.A) for beta-defensin 2.

The three-dimensional structures of both sea bass beta-defensins were predicted using the SWISS-MODEL server (Figure 3). The beta-defensin 1 model was predicted using as a template the NMR structure of human beta-defensin 6. According to the predicted tertiary structure, sea bass beta-defensin 1 folds into three antiparallel beta-sheets, with some disordered areas and an extra alpha-helix in the N-terminal. These secondary structures are stabilized by the disulphide bonds formed between Cys31–Cys60, Cys38–Cys54 and Cys42–Cys61. The beta-defensin 2 model was predicted using as templates the crystal structure of human beta-defensin 4 and a NMR structure of oyster big defensin. This peptide presents disordered areas, and the N-terminal is not predicted to fold into an alpha-helix. Still, beta-defensin 2 presents the three antiparallel beta-sheets, with the six cysteines forming the disulphide bonds between Cys30–Cys58, Cys36–Cys52 and Cys40–Cys59.

### 2.3. Genomic Organization

**Figure 4:**
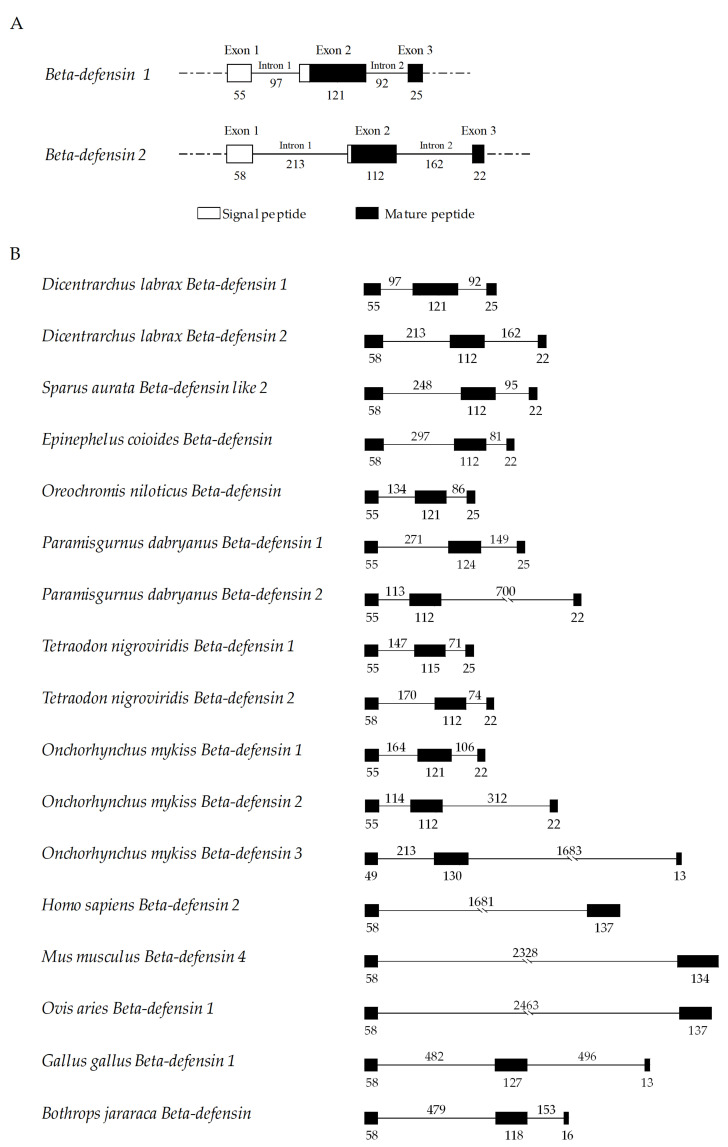
Genomic organization of sea bass beta-defensin genes. (**A**) Exon/intron diagram of sea bass beta-defensin genes. (**B**) Comparative view with other vertebrate beta-defensins. Exons are shown as boxes and introns as solid lines, with sizes of exons/introns in base pairs.

Sea bass beta-defensin genes are constituted by three exons and two introns. The beta-defensin 1 gene consists of exons of 55, 121 and 25 bp and introns of 97 and 92 bp. The beta-defensin 2 gene presents exons of 58, 112 and 22 bp and introns of 213 and 162 bp (Figure 4A). The signal peptides are encoded by exons 1 and 2, and the mature peptides are encoded by exons 2 and 3. Both genes present exons with similar sizes, exon 2 being the longest exon and exon 3 the smallest one. The most significant differences are observed between introns, with beta-defensin 2 presenting longer introns when compared to beta-defensin 1. Sea bass beta-defensin genes were compared with the ones of other vertebrates (Figure 4B), with fish species presenting a similar structure of three exons/two introns, as well as birds and reptiles. Mammalian beta-defensins present only two exons, with similar sizes to fish exons 1 and 2, and one intron, that is much larger than fish introns.

### 2.4. Sequence Comparison, Phylogenetic and Syntenic Analysis

**Figure 5:**
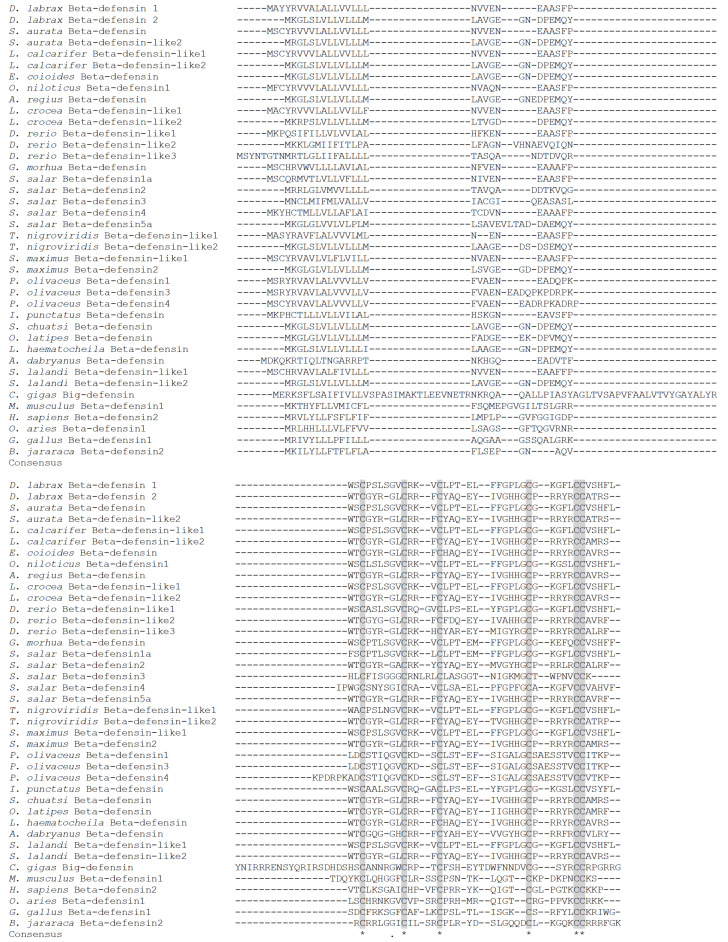
Alignment of sea bass beta-defensins with peptides from other vertebrate species. Cysteines are shaded gray and are denoted by (*) and semi-conserved substitutions by (.).

Sequence comparison with other vertebrate beta-defensins showed a high degree of identity within each subfamily. Beta-defensin 1 shares an identity with other vertebrate peptides between 28% and 97% and beta-defensin 2 between 33% and 99% (Supplementary Figure S1). The characteristic six cysteines are retained in all peptides at conserved positions. Furthermore, beta-defensin 2 shares a similar motif between other type 2 beta-defensins, namely the CPRR(Y/L/F)K motif that is found between Cys52 and Cys58. (Figure 5).

**Figure 6:**
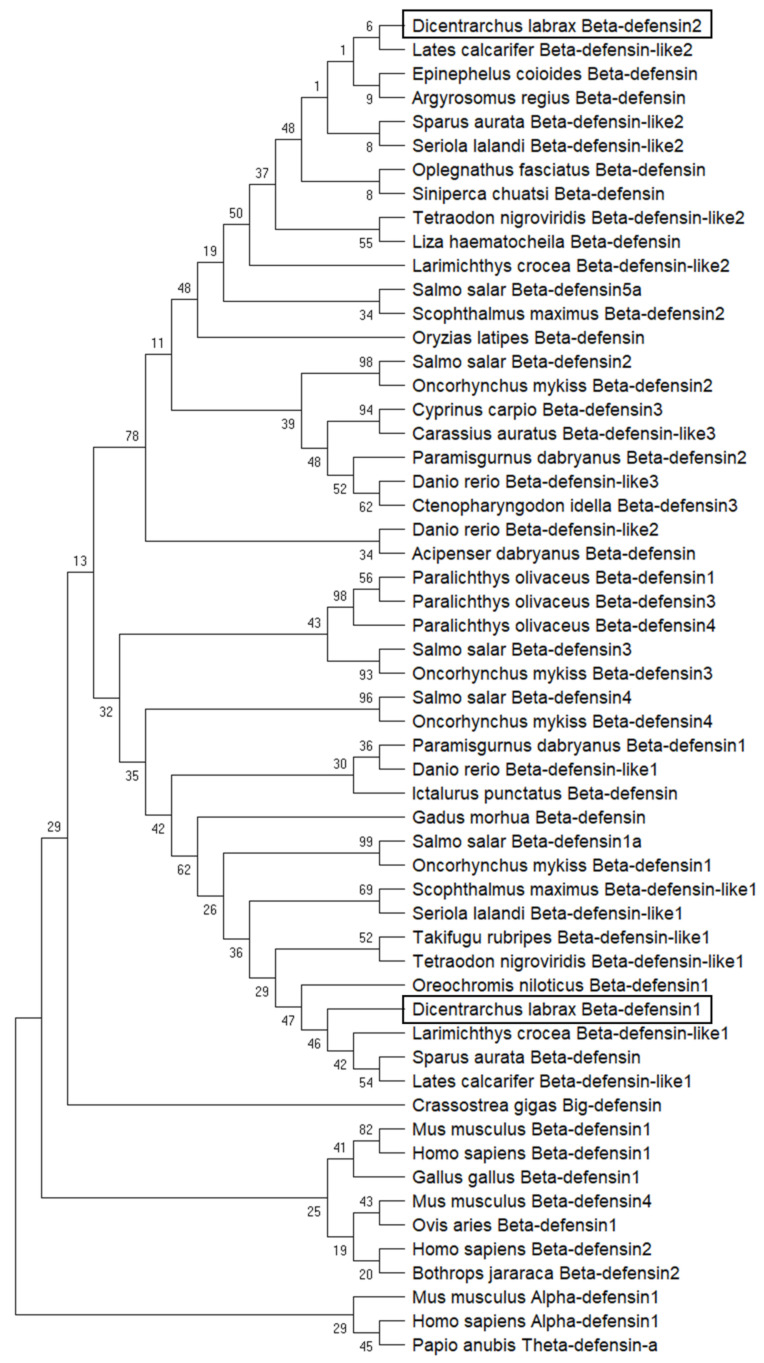
Phylogenetic analysis of beta-defensin peptides. The evolutionary history was inferred by using the Maximum Likelihood method and JTT matrix-based model [32]. The bootstrap consensus tree inferred from 1000 replicates is taken to represent the evolutionary history of the taxa analyzed [33]. Branches corresponding to partitions reproduced in less than 50% bootstrap replicates are collapsed. The percentage of replicate trees in which the associated taxa clustered together in the bootstrap test (1000 replicates) are shown next to the branches [33]. Initial tree(s) for the heuristic search were obtained automatically by applying Neighbor-Join and BioNJ algorithms to a matrix of pairwise distances estimated using the JTT model, and then selecting the topology with superior log likelihood value. This analysis involved 56 amino acid sequences. All positions containing gaps and missing data were eliminated (complete deletion option). There was a total of 37 positions in the final dataset. Evolutionary analyses were conducted in MEGA X [34].

Phylogenetic analysis separated fish beta-defensins from peptides belonging to other vertebrate species and *Crassostrea gigas* big defensin (Figure 6). Within fish defensins, two larger clades can be observed: the first is a heterogeneous group composed by two smaller clusters, one including all beta-defensins type 2 and also beta-defensin 5a from *S. salar*, and the other group includes all beta-defensins type 3, particularly from Cyprinid species, and beta-defensin 2 from Salmoniformes. The second clade includes all beta-defensins type 1 from the different species, as well as peptides belonging to *P. olivaceus* and also beta-defensins 3 and 4 from Salmonid species, that are included in two small groups. In both clades, a division between the different species is clear: Perciformes cluster with species belonging to Tetraodontiformes, Pleuronectiformes (with the exception of *P. olivaceus*), Centrarchiformes or Carangiformes, being separated from Salmoniformes, Cypriniformes or Gadiformes.

**Figure 7:**
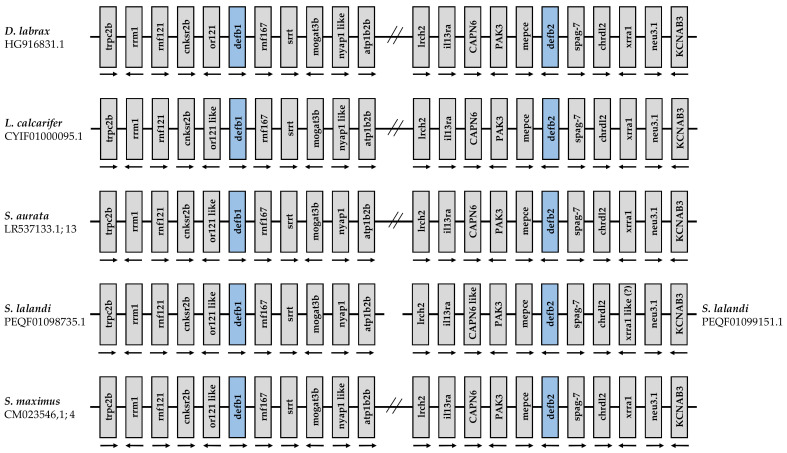
Analysis of beta-defensin loci in the European sea bass (*D. labrax*, location HG916831.1), Barramundi perch (*L. calcarifer*, location CYIF01000095.1), Gilthead seabream (*S. aurata*, location LR537133.1; 13), Yellowtail amberjack (*S. lalandi*, locations PEQF01098735.1 and PEQF01099151.1 for beta-defensins 1 and 2, respectively) and Turbot (*S. maximus*, location CM023546.1; 4). Analysis was performed using sequences available in the Ensembl genome browser 103, and the corresponding accession numbers of each gene are described in Table S2. *S. lalandi xrra1*: incomplete gene.

The genomic location of sea bass beta-defensins was analyzed and compared with two perciform species (*Lates calcarifer* and *Sparus aurata*) and two other species, *Seriola lalandi* and *Scophthalmus maximus*, that belong to the Carangiformes and Pleuronectiformes orders, respectively (Figure 7). Based on available date from the Ensembl database, both sea bass defensins are expected to be located in the same chromosome, as well as *L. calcarifer*, *S. aurata* and *S. maximus*. On the contrary, defensins of *S. lalandi* were found in different locations. Still, synteny analysis reveals a high degree of conservation of these genetic loci, with all of the genes found in the vicinity of sea bass beta-defensins also being found in the other species analyzed.

### 2.5. Basal Expression of Sea Bass Beta-Defensins

**Figure 8:**
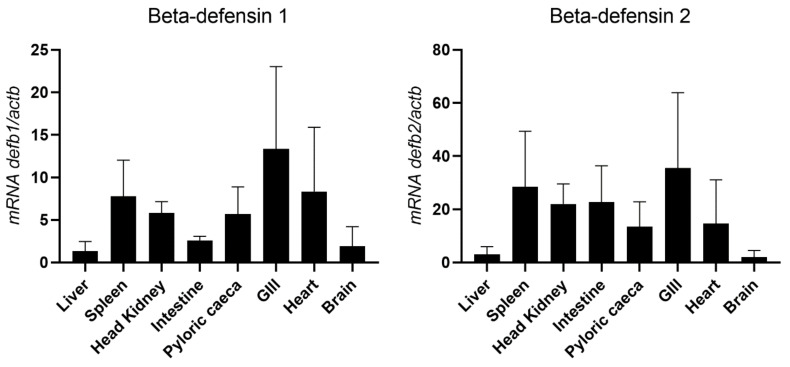
Basal expression of beta-defensin genes in different organs of healthy sea bass, measured by real-time PCR. Each sample was normalized to beta actin (*actb*) calculated by the comparative CT method (2^−ΔΔCT^). Values are presented as means ± standard deviation (S.D.) (*n* = 5).

Different tissues of healthy sea bass were used to evaluate the constitutive expression of beta-defensins, namely the liver, spleen, head kidney, intestine, pyloric caeca, gill, heart and brain (Figure 8). Both defensins were detected in all tissues analyzed, with the spleen, head kidney and gills being the tissues with the highest relative basal expression of both beta-defensins. For beta-defensin 1, basal expression is also high in the pyloric caeca and heart, followed by the intestine, with the lowest expression being observed in the brain and liver. For beta-defensin 2, a high expression is also observed in the intestine, followed by the heart and pyloric caeca, with the liver and brain being the tissues with the lowest basal expression.

## 3. Discussion

In the present study, we focused on the molecular characterization of two beta-defensin family members in the European sea bass (*Dicentrarchus labrax*). This fish species presents two different peptides, sharing similar features with other fish beta-defensins, including the six cysteines at conserved positions, folding into three antiparallel beta-sheets, and an overall cationic net charge [19].

Some invertebrate species express a group of peptides called big defensins, formed by a N-terminal hydrophobic domain, mainly constituted by alpha-helices, and a C-terminal beta-defensin-like domain that includes three disulfide bridges that are linked in the pattern C1–C5, C2–C4 and C3–C6, resulting in three beta-sheets [35,36,37,38]. In vertebrate species, three different classes of defensins are found, namely alpha-, beta- and the cyclic theta-defensins, with beta-defensins being found widespread throughout different species [2,8,13,39], while alpha- and theta-defensins are more restricted to mammals [40]. Theta-defensins are even more exclusive, being a result of two truncated alpha-defensins and found only in some primate species, while in humans they became inactivated, due to mutations that resulted in a premature stop codon [10,11,41]. The presence of beta-defensins even in more primitive vertebrates, such as fish or reptiles, may indicate that these particular peptides constitute a more ancient type [40]. Beta-defensins and the C-terminal domain of big defensins share similar features, including the genomic organization, secondary structures and identical cysteine bridges [42]. Thus, beta-defensins likely arose from these big defensins of invertebrate species, through processes of intronization of exonic sequences or exon shuffling, leading to the loss of the amino-terminal domain and the appearance of an ancestral vertebrate beta-defensin, with a two exon/one intron organization [12,42]. Then, species-specific events of intron insertions have led to the appearance of the three exon/two intron structure observed in birds and fish, while mammals retained the original organization [3,12]. Later, after divergence of mammals from other species, extensive events of local duplication originated several clusters of defensins in mammals, and also led to the appearance of alpha- and theta-defensins [13,39,43].

Beta-defensins are present in fish in a varied number and are highly divergent from the mammalian counterparts in terms of sequence and genomic organization. In sea bass and other Perciformes (and also some Pleuronectiformes, Carangiformes or Cichliformes), up to two peptides have been isolated or annotated in genome databases, and belong to beta-defensin types 1 and 2, with no members of other defensin subfamilies so far being isolated in these species. Beta-defensin types 1 and 2 present a low identity, but retain the six cysteines and the triple-stranded beta-sheet structure. However, within each group, beta-defensins share high identity scores, and the genetic loci of these species are well conserved, with many genes surrounding beta-defensins being found in different fish. Other species, particularly the ones belonging to Salmoniformes, express up to seven different defensins [3,12,44]. Sea bass beta-defensins 1 and 2 are more related to beta-defensins 1 and 5 from Atlantic salmon, while the other Salmonid peptides are separated in different branches. Synteny studies also reinforce the relationship between sea bass peptides and these salmonid defensins, as some genes found in the vicinity of sea bass defensins are also found next to Salmonid defensins 1 and 5 [12]. During evolution, Teleost fish have faced three rounds of whole genome duplications (3WGD or fish-specific WGD), with the salmonid lineage presenting an additional WGD (4WGD or salmonid-specific WGD) [45,46]. These events of genome duplications are often correlated with the diversity of species found among the Teleostean [47]. Many of these duplicated genes were lost during evolutionary processes, while others were retained and even gained novel functions in a lineage-specific manner [48,49]. Beta-defensin genes in salmonids derived from common ancestry and then expanded, after these events of WGDs [12], and diverged from other species, including the European sea bass. While in Salmonid species several beta-defensins can be found, in Perciformes and perhaps other related species, they turned into a more restricted group, with other families of AMPs being more diversified, such as hepcidins or piscidins [50,51].

Three dimensional models of sea bass beta-defensins were predicted using the Swiss model server, with the available NMR or crystal structures of beta-defensins from humans [52,53] and the beta-defensin-like domain of oyster big-defensin [35]. Both sea bass defensins are predicted to fold into three antiparallel beta-sheets, stabilized by the disulphide bonds between cysteines, with beta-defensin 1 presenting an extra alpha-helix at the N-terminal. This alpha-helix is also observed in other fish and non-fish beta-defensins. In fish, it seems that the presence of this secondary structure is not exclusive to any beta-defensin type. The alpha-helix was predicted in type 1 beta-defensins of zebrafish, Atlantic cod and blunt snout bream (*Megalobrama amblycephala*) [2,4,28], but it was not found in beta-defensin 1 of Nile tilapia or channel catfish [5,18]. On the contrary, in soiny mullet (*Liza haematocheila*) and turbot (*S. maximus*), the authors describe the presence of an alpha-helix at the N-terminal of beta-defensins characterized in these species, both belonging to the type 2 subfamily [17,54]. In other vertebrate species, a similar folding is observed in different beta-defensins, including human and mouse beta-defensins [52,55,56,57,58,59]. Aside the alpha-helix, the three antiparallel beta-sheets were predicted in sea bass beta-defensin 1, through binding between Cys31–Cys60, Cys38–Cys54 and Cys42–Cys61. For sea bass beta-defensin 2, using the models available in the Swiss model, no alpha-helix was observed. Still, the three antiparallel beta-sheets are present, with the cysteines being linked between Cys30–Cys58, Cys36–Cys52 and Cys40–Cys59. This pattern of cysteine binding is a hallmark of beta-like defensins. Alpha-defensins, on the contrary, are usually smaller and lack the presence of alpha-helices, and, although they also consist in a triple-stranded beta-sheet, their cysteines are connected in the Cys1–Cys6, Cys2–Cys4, Cys3–Cys5 pattern [1,60]. Theta-defensins present a circular structure, without a free N- or C-terminal, as a result of cyclization of two 9-amino-acid segments of alpha-defensin-like molecules [10]. Beta-defensin 2 also presents the CPRRYK basic motif, between Cys4 and Cys5, which is found in all fish beta-defensins type 2 and 3, with the exception of *P. olivaceus*. This motif is also present in human beta-defensin 2, although in a different loop, between Cys3 and Cys4 [61]. The presence of this conserved motif in phylogenetic distant organisms may suggest a common functional role [2].

The tertiary structure of beta-defensins is similar to what is verified for chemokines, although their amino acid sequences share a reduced identity [60,62]. In fact, human and mice beta-defensins present chemotactic activity towards diverse leukocytes, using different chemokine receptors [29,63]. Fish beta-defensins are also chemotactic for different leukocytes, although the exact mechanism remains undetermined [14,28]. Still, authors observed that head kidney leukocytes were attracted to the recombinant seabream defensin 1, but the same was not observed using recombinant human peptides, suggesting a certain degree of selection by fish receptors [14]. Furthermore, the N-terminal portion of blunt snout bream defensin exerts an increased chemotactic activity of head kidney leukocytes, when compared to the C-terminal portion, suggesting that the N-terminal of beta-defensin might be important for this function [28]. Fish beta-defensins are also known to present antibacterial and antiviral activities [4,27,54,64]. The mechanism of action of AMPs relies on an initial interaction between the peptide and bacterial membrane [65]. Particularly for defensins, previous authors suggest that the alpha-helix might be helpful in the interaction of beta-defensin with the bacterial cell wall [66]. Indeed, synthetic and recombinant beta-defensin 2 from large yellow croaker and turbot impaired membrane morphology were recently described, leading to a severe membrane damage [27,54]. Given the similarities between these peptides and sea bass beta-defensins, particularly defensin type 1, we speculate that they may also be involved in such functions, although further studies are necessary to uncover the possible antimicrobial and chemotactic activities of each sea bass defensin.

Sea bass beta-defensins were detected in all tissues analyzed, with an overall predominance of beta-defensin 2. Both genes are highly expressed in the gills, head kidney and spleen, with a low expression in the liver. In other fish species, beta-defensins are also highly expressed in tissues including the skin, gills, head kidney or spleen [5,14,15,20]. On the contrary, in turbot and grouper, beta-defensin 2 shows high basal expression levels in the skin and gills, but also in the liver, with a low expression in the spleen and kidney [26,54]. In salmonids species, the several defensins can present a different pattern of expression, depending on the gene and tissue analyzed, with some defensins being more expressed in other tissues, including the liver [3,12]. In mammals, beta-defensins can also be found in the skin and mucosal surfaces, acting as a first line of defense [67,68]. Assuming the antimicrobial and immunomodulatory roles of beta-defensins, it is not surprising to find a high constitute expression in tissues exposed to the external environment, as well as in fish central immune tissues. Still, beta-defensins show different patterns of basal expression depending on the species, tissue and gene analyzed.

## 4. Materials and Methods

### 4.1. Animals

Healthy European sea bass (*D. labrax*), with an average weight of 30 g, were provided by a commercial fish farm (Sonríonansa, Pesués, Cantabria, Spain) [50]. Prior to the experiments, fish were acclimated for 30 days to the fish-holding facilities of the Instituto de Ciências Biomédicas Abel Salazar (ICBAS), Porto. Fish were kept in 110 L recirculating sea water (28‰ salinity) tanks at 22 ± 1 °C, with a 13 h light/11 h dark cycle and fed daily ad libitum with commercial fish feed. Before each treatment, fish were anesthetized with ethylene glycol monophenyl ether (2-phenoxyethanol, 0.3 mL/L; Merck, Algés, Portugal).

### 4.2. Isolation of Sea Bass Beta-Defensins

Pairs of oligonucleotide PCR primers were designed according to conserved regions of beta-defensins’ mRNA sequences from sea bass and other fish species, with sea bass expressed sequence tags (ESTs) and whole-genome shotgun sequences (WGSS) available in the National Center for Biotechnology Information nucleotide database (http://www.ncbi.nlm.nih.gov, accessed on April 2021) and Ensembl genome browser 103 (http://www.ensembl.org, accessed on April 2021). cDNA preparations from the whole intestine, kidney and gill were used in PCR amplifications [50,69]. PCR products were run on 1% agarose gels, and then relevant fragments were purified with the NZYGelpure kit (NZYtech, Lisbon, Portugal), cloned into pCR 2.1-TOPO vectors, propagated in One Shot Mach1-T1R competent cells (Invitrogen, Life Technologies, Carlsbad, CA) and sent for sequencing (GATC, A Eurofins Genomics Company, Ebersberg, Germany). Both strands were sequenced, and chromatograms were analyzed in FinchTV (Geospiza, Seattle, WA, USA) and assembled using Multalin (http://multalin.toulouse.inra.fr/multalin/, accessed on April 2021).

### 4.3. Modeling of Sea Bass Beta-Defensin Peptides

Prediction of the three-dimensional structures of sea bass beta-defensins was performed by protein homology detection/modelling, using the SWISS-MODEL server (https://swissmodel.expasy.org/, accessed on April 2021). Templates were chosen using the best GMQE (Global Model Quality Estimation) and QMEAN (Qualitative Model Energy Analysis) estimations, as well as the highest sequence identity and coverage between templates and sea bass beta-defensins. Human beta-defensin 6 (2lwl.1.A) [52] NMR structure was used as the template for sea bass beta-defensin 1 modeling, while the crystal structure of human beta-defensin 4 (5ki9.1.A) [53] and NMR structure of oyster (C. gigas) big defensin (6qbk.1.A) [35] were used for sea bass beta-defensin 2. Molecular graphic images were obtained using the Polyview-3D webserver (http://polyview.cchmc.org/polyview3d.html, accessed on April 2021) [70].

### 4.4. Genomic Organization

Genomic DNA was isolated from sea bass red blood cells, using the NZY Blood gDNA Isolation kit (NZYtech), as previously described [50,69]. Quantification was performed using a NanoDrop 1000 spectrophotometer (Thermo Fisher Scientific, Waltham, MA, USA), and quality was checked by agarose gel electrophoresis. Two micrograms of genomic DNA were amplified by PCR with the primers based on the previously obtained cDNA sequences, with the following cycling profile: 94 °C for 5 min, 30 cycles of 94 °C for 60 s, 59 °C for 60 s, 72 °C for 60 s and a final step of 72 °C for 5 min. Several PCR products were purified, cloned and sent for sequencing. Comparisons were made between genomic DNA and cDNA to evaluate the similarity of the coding regions and to identify intron/exon boundaries. A comparison between the genomic sequences of sea bass beta-defensins with those of other vertebrate species was also made, using the sequences identified with the GenBank and Ensembl accession numbers described in Table S1.

### 4.5. Alignment, Phylogenetic and Syntenic Analysis

Alignments of the amino acid sequences of beta-defensin predicted proteins were performed using MUSCLE from MEGA X [34]. A phylogenetic tree was constructed using the Maximum Likelihood method, with the Jones–Taylor–Thornton (JTT) model, Nearest-Neighbor-Interchange heuristic model, complete deletion of gaps and 1000 bootstrap replications. Sequences used for comparisons and phylogenetic trees and their accession numbers are shown in Table S2.

Synteny analysis between the European sea bass genome and other species was conducted using information available in Ensembl genome browser 103. Orthology/paralogy relationships were derived from the ortholog/paralog prediction function of the Ensembl website. The accession numbers of genes studied are shown in Table S3.

### 4.6. RNA Isolation and cDNA Synthesis

Total RNA was isolated from tissues with the NZY Total RNA Isolation Kit (NZYTech), with the optional on-column DNase treatment, according to the manufacturer’s instructions [50,69]. Total RNA quantification was performed using a NanoDrop 1000 spectrophotometer (Thermo Fisher Scientific), and quality was assessed by running the samples in an Experion Automated Electrophoresis Station (Bio-Rad, Hercules, CA, USA). For all samples, 2.5 µg of each were converted to cDNA using the NZY First-Strand cDNA Synthesis Kit (NZYTech), according to the manufacturer’s protocol.

### 4.7. Basal Expression of Sea Bass Beta-Defensins

Several tissues from five healthy sea bass were collected for RNA isolation and cDNA synthesis, as previously described [50,69]. Relative levels of beta-defensin mRNAs were quantified by real-time PCR analysis using an CFX96 Real-Time PCR Detection System (Bio-Rad). Pairs of primers used for the reactions were designed according to our beta-defensin sequences and are as follows: defb1-For 5′-ATGGCTTATTATCGTGTGGTTG-3′/defb1-Rev 5′-TCATAGAAAATGAGACA-CACAGC-3′ for beta-defensin 1 and defb2-For 5′-ATGAAGGGACTGAGCTTGGTT-3′/defb2-Rev 5′-CTAAGAACGTGTAGCACAGC-3′ for beta-defensin 2. One µL of each cDNA sample was added to a reaction mix containing 10 µL iTaq Universal SYBR Green Supermix (Bio-Rad), 7 µL of ddH20 and 250 nM of each primer, making a total volume of 20 µL per reaction. The cycling profile was as follows: 95 °C for 3.5 min, 40 cycles of 95 °C for 20 s and 59 °C for 20 s. Samples were prepared in duplicates, a melting curve was generated for every PCR product to confirm the specificity of the assays, and a dilution series was prepared to check the efficiency of the reactions. Beta-actin (actb) was used as the housekeeping gene (primers actb-For 5′-CAGAAGGACAGCTACGT-3′/actb-Rev 5′-GTCATCTTCTC-CCTGTTGGC-3′). The comparative CT method (2^−ΔΔCT^ method) based on cycle threshold values was used to analyze gene expression levels. Graphics were generated using GraphPad Prism 9 (GraphPad Software, San Diego, CA, USA).

## 5. Conclusions

In summary, we identified two beta-defensins in the European sea bass, with high similarities with other fish peptides in terms of sequence, cysteine position and tertiary structure. Defensins are the most studied group of AMPs, with many fish and non-fish peptides already characterized. However, reports addressing the beta-defensin family in sea bass are still lacking. Further studies are necessary to understand the functions of sea bass beta-defensins, including their antimicrobial and immunomodulatory functions, and how these genes are modulated during infection. It is known that defensins are involved in several immune roles [19], and, as such, an in-depth knowledge of sea bass AMPs might be helpful in the development of novel prophylactic or therapeutic compounds to be used in the production of sea bass.

## Data Availability

Data is contained within the article or Supplementary Materials.

## Supplementary Materials

The following are available online at https://www.mdpi.com/article/10.3390/ph14060566/s1, Figure S1: Identity scores between vertebrate beta-defensins; Table S1: Accession numbers of vertebrate beta-defensin genes; Table S2: Accession numbers of defensins used in the alignment and phylogenetic analysis; Table S3: Accession numbers of genes used in the synteny analysis of beta-defensins 1 and 2.
