# Supplementary material for: Antimicrobial Peptides: Identification of two Beta-Defensins in a Teleost Fish, the European Sea Bass (Dicentrarchus labrax)
Source: Pharmaceuticals (Basel). 2021 Jun 14;14(6):566. doi: 10.3390/ph14060566 (PMC8231796; doi:10.3390/ph14060566)
Supplement: Supplementary file 1 [file pharmaceuticals-14-00566-s001.zip › Supplementary Figure1.pdf]

	D. labrax β-defensin1	*D. labrax* β-defensin2	*S. aurata* β-defensin	*S. aurata* β-defensin-like2	*L. calcarifer* β-defensin-like1	*L. calcarifer* β-defensin-like2	*E. coioides* β-defensin	*O. niloticus* β-defensin1	*A. regius* β-defensin	*L. crocea* β-defensin-like1	*L. crocea* β-defensin-like2	*D. rerio* β-defensin-like1	*D. rerio* β-defensin-like2	*D. rerio* β-defensin-like3	*G. morhua* β-defensin	*S. salar* β-defensin1a	*S. salar* β-defensin2	*S. salar* β-defensin3	*S. salar* β-defensin4	*S. salar* β-defensin5a	*T. nigroviridis* β-defensin-like1	*T. nigroviridis* β-defensin-like2	*S. maximus* β-defensin-like1	*S. maximus* β-defensin2	*P. olivaceus* β-defensin1	*P. olivaceus* β-defensin3	*P. olivaceus* β-defensin4	*I. punctatus* β-defensin	*S. chuatsi* β-defensin	*O. latipes* β-defensin	*L. haematocheila* β-defensin	*A. dabryanus* β-defensin	*S. lalandi* β-defensin-like1	*S. lalandi* β-defensin-like2	*M. musculus* β-defensin1	*H. sapiens* β-defensin2	*O. aries* β-defensin1	*G. gallus* β-defensin1	*B. jararaca* β-defensin2
D. labrax β-defensin1	100%																																						
D. labrax β-defensin2	46%	100%																																					
S. aurata β-defensin	97%	46%	100%																																				
S. aurata β-defensin-like2	47%	99%	47%	100%																																			
L. calcarifer β-defensin-like1	97%	46%	100%	47%	100%																																		
L. calcarifer β-defensin-like2	46%	99%	46%	98%	46%	100%																																	
E. coioides β-defensin	46%	98%	46%	97%	46%	98%	100%																																
O. niloticus β-defensin1	92%	44%	94%	46%	94%	44%	44%	100%																															
A. regius β-defensin	44%	98%	44%	97%	44%	98%	98%	43%	100%																														
L. crocea β-defensin-like1	97%	46%	98%	47%	98%	46%	46%	93%	44%	100%																													
L. crocea β-defensin-like2	48%	92%	48%	91%	48%	92%	92%	47%	92%	48%	100%																												
D. rerio β-defensin-like1	80%	41%	80%	41%	80%	41%	41%	78%	40%	79%	43%	100%																											
D. rerio β-defensin-like2	39%	69%	39%	68%	39%	69%	70%	40%	70%	39%	69%	39%	100%																										
D. rerio β-defensin-like3	41%	61%	41%	62%	41%	61%	60%	42%	60%	40%	60%	37%	54%	100%																									
G. morhua β-defensin	83%	43%	87%	44%	87%	43%	43%	81%	42%	84%	46%	76%	40%	41%	100%																								
S. salar β-defensin1a	87%	46%	89%	47%	89%	46%	46%	83%	44%	87%	48%	76%	38%	40%	83%	100%																							
S. salar β-defensin2	49%	74%	49%	76%	49%	74%	73%	49%	73%	48%	74%	43%	61%	74%	47%	49%	100%																						
S. salar β-defensin3	41%	47%	41%	47%	41%	47%	47%	41%	46%	41%	46%	41%	39%	37%	40%	42%	47%	100%																					
S. salar β-defensin4	57%	48%	57%	47%	57%	48%	48%	56%	48%	58%	47%	56%	42%	33%	57%	53%	44%	43%	100%																				
S. salar β-defensin5a	41%	84%	41%	83%	41%	84%	84%	42%	86%	41%	82%	40%	72%	58%	39%	41%	70%	41%	43%	100%																			
T. nigroviridis β-defensin-like1	88%	42%	87%	43%	87%	42%	42%	84%	41%	87%	44%	74%	38%	40%	76%	79%	48%	39%	54%	39%	100%																		
T. nigroviridis β-defensin-like2	44%	93%	44%	92%	44%	92%	91%	46%	91%	44%	88%	41%	69%	61%	42%	44%	74%	47%	49%	84%	42%	100%																	
S. maximus β-defensin-like1	90%	42%	93%	43%	93%	42%	42%	90%	41%	91%	44%	77%	40%	41%	83%	84%	47%	38%	53%	41%	82%	43%	100%																
S. maximus β-defensin2	46%	96%	46%	94%	46%	97%	94%	44%	94%	46%	91%	41%	70%	61%	43%	46%	74%	46%	47%	87%	42%	90%	42%	100%															
P. olivaceus β-defensin1	58%	41%	60%	42%	60%	40%	40%	59%	39%	59%	42%	48%	36%	37%	56%	54%	42%	41%	40%	37%	53%	43%	59%	40%	100%														
P. olivaceus β-defensin3	50%	39%	52%	40%	52%	38%	38%	51%	38%	51%	38%	40%	33%	32%	48%	47%	38%	34%	36%	36%	46%	40%	51%	38%	91%	100%													
P. olivaceus β-defensin4	46%	33%	49%	34%	49%	32%	32%	48%	32%	48%	32%	36%	27%	27%	46%	43%	33%	30%	34%	28%	41%	34%	48%	32%	79%	81%	100%												
I. punctatus β-defensin	66%	49%	66%	48%	66%	49%	49%	67%	49%	66%	48%	71%	44%	36%	60%	63%	46%	44%	64%	46%	62%	49%	62%	48%	40%	36%	31%	100%											
S. chuatsi β-defensin	46%	99%	46%	98%	46%	100%	98%	44%	98%	46%	92%	41%	69%	61%	43%	46%	74%	47%	48%	84%	42%	92%	42%	97%	40%	38%	32%	49%	100%										
O. latipes β-defensin	46%	90%	46%	89%	46%	91%	89%	46%	89%	46%	86%	42%	69%	64%	43%	46%	74%	48%	47%	83%	43%	88%	43%	91%	42%	41%	36%	50%	91%	100%									
L. haematocheila β-defensin	44%	96%	44%	94%	44%	96%	98%	46%	96%	44%	90%	41%	71%	60%	42%	44%	72%	47%	48%	84%	42%	91%	43%	92%	41%	39%	33%	50%	96%	88%	100%								
A. dabryanus β-defensin	47%	57%	47%	58%	47%	57%	56%	46%	56%	47%	59%	44%	53%	56%	48%	46%	59%	38%	38%	53%	47%	57%	47%	57%	41%	33%	28%	38%	57%	56%	56%	100%							
S. lalandi β-defensin-like1	91%	43%	94%	44%	94%	43%	43%	89%	42%	92%	46%	77%	41%	42%	87%	84%	48%	39%	54%	39%	82%	42%	93%	43%	59%	51%	48%	62%	43%	43%	42%	47%	100%						
S. lalandi β-defensin-like2	47%	98%	47%	99%	47%	98%	98%	46%	98%	47%	92%	41%	69%	62%	44%	47%	76%	47%	47%	84%	43%	91%	43%	94%	41%	39%	33%	48%	98%	89%	96%	58%	44%	100%					
M. musculus β-defensin1	28%	34%	28%	33%	28%	34%	34%	28%	34%	29%	33%	27%	30%	23%	28%	28%	31%	32%	34%	32%	27%	34%	28%	34%	30%	30%	27%	33%	34%	33%	34%	27%	28%	33%	100%				
H. sapiens β-defensin2	36%	42%	36%	43%	36%	42%	42%	37%	42%	37%	40%	34%	37%	33%	36%	34%	40%	39%	39%	39%	34%	41%	36%	42%	34%	32%	28%	40%	42%	41%	42%	33%	36%	43%	47%	100%			
O. aries β-defensin1	36%	41%	36%	42%	36%	41%	41%	37%	41%	36%	40%	34%	37%	31%	37%	37%	39%	39%	36%	39%	36%	41%	38%	42%	41%	40%	33%	44%	41%	41%	42%	34%	36%	42%	43%	56%	100%		
G. gallus β-defensin1	43%	44%	43%	46%	43%	44%	44%	43%	44%	42%	43%	41%	38%	36%	40%	42%	43%	41%	40%	40%	41%	43%	41%	44%	40%	37%	30%	43%	44%	43%	44%	34%	42%	46%	47%	46%	46%	100%	
B. jararaca β-defensin2	41%	43%	41%	42%	41%	43%	43%	40%	42%	41%	42%	42%	39%	38%	40%	41%	44%	39%	39%	41%	39%	42%	41%	43%	37%	31%	26%	43%	43%	44%	43%	36%	41%	42%	40%	51%	44%	49%	100%

Supplementary Figure 1. Identity scores between vertebrate beta-defensins. Percentages were determined using the SIAS (Sequence Identity And Similarity) server (http://imed.med.ucm.es/Tools/sias.html), with BLOSUM62 scoring matrix and considering the length of multiple sequence alignment.
