# Supplementary material for: Antimicrobial Peptides: Identification of two Beta-Defensins in a Teleost Fish, the European Sea Bass (Dicentrarchus labrax)
Source: Pharmaceuticals (Basel). 2021 Jun 14;14(6):566. doi: 10.3390/ph14060566 (PMC8231796; doi:10.3390/ph14060566)
Supplement: Supplementary file 1 [file pharmaceuticals-14-00566-s001.zip › Supplementary Table1.pdf]

Table S1 Accession numbers of vertebrate beta-defensin genes.

Species	Order	Gene	Accession number
Dicentrarchus labrax	Perciformes	Beta-defensin 1	CBXY010003646
Dicentrarchus labrax	Perciformes	Beta-defensin 2	CBXY010003659
Sparus aurata	Perciformes	Beta-defensin-like 2	ENSSAUG00010026539
Epinephelus coioides	Perciformes	Beta-defensin	JN698964
Oreochromis niloticus	Cichliformes	Beta-defensin 1	KJ577575
Paramisgurnus dabryanus	Cypriniformes	Beta-defensin 1	KC494302
Paramisgurnus dabryanus	Cypriniformes	Beta-defensin 2	KC261302
Tetraodon nigroviridis	Tetraodontiformes	Beta-defensin-like 1	BN000873
Tetraodon nigroviridis	Tetraodontiformes	Beta-defensin-like 2	BN000874
Onchorhynchus mykiss	Salmoniformes	Beta-defensin 1	FN545575
Onchorhynchus mykiss	Salmoniformes	Beta-defensin 2	FN545576
Onchorhynchus mykiss	Salmoniformes	Beta-defensin 3	FN545577
Homo sapiens	Primates	Beta-defensin 2	AF040153
Mus musculus	Rodentia	Beta-defensin 4	AF288371
Ovis aires	Artiodactyla	Beta-defensin 1	BK010312
Gallus gallus	Galliformes	Beta-defensin 1	AY621316
Bothrops jararaca	Squamata	Beta-defensin	KC117163
