# Supplementary material for: Antimicrobial Peptides: Identification of two Beta-Defensins in a Teleost Fish, the European Sea Bass (Dicentrarchus labrax)
Source: Pharmaceuticals (Basel). 2021 Jun 14;14(6):566. doi: 10.3390/ph14060566 (PMC8231796; doi:10.3390/ph14060566)
Supplement: Supplementary file 1 [file pharmaceuticals-14-00566-s001.zip › Supplementary Table2.pdf]

Table S2 Accession numbers of defensins used in the alignment and phylogenetic analysis.

Species		Gene	Accession number
Sparus aurata	Perciformes	Beta-defensin	FM158209
Sparus aurata	Perciformes	Beta-defensin-like 2	ENSSAUT00010069764.1
Epinephelus coioides	Perciformes	Beta-defensin	AET25528
Argyrosomus regius	Perciformes	Beta-defensin	ASW20415
Lates calcarifer	Perciformes	Beta-defensin-like 1	ENSLCAT00010056645.1
Lates calcarifer	Perciformes	Beta-defensin-like 2	ENSLCAT00010013452.1
Larimichthys crocea	Perciformes	Beta-defensin-like 1	ENSLCRT00005005151.1
Larimichthys crocea	Perciformes	Beta-defensin-like 2	ENSLCRT00005035395.1
Oreochromis niloticus	Cichliformes	Beta-defensin 1	AGW83444
Paramisgurnus dabryanus	Cypriniformes	Beta-defensin 1	AGK65596
Paramisgurnus dabryanus	Cypriniformes	Beta-defensin 2	AGH10110
Cyprinus carpio L.	Cypriniformes	Beta-defensin 3	AGZ03658
Danio rerio	Cypriniformes	Beta-defensin-like 1	NP_001075022
Danio rerio	Cypriniformes	Beta-defensin-like 2	NP_001075023
Danio rerio	Cypriniformes	Beta-defensin-like 3	NP_001075024
Ctenopharyngodon idella	Cypriniformes	Beta-defensin 3	AQY18997
Carassius auratus	Cypriniformes	Beta-defensin-like 3	ENSCART00000074063.1
Gadus morhua	Gadiformes	Beta-defensin	AEB69787
Salmo salar	Salmoniformes	Beta-defensin 1a	QIX04703
Salmo salar	Salmoniformes	Beta-defensin 2	QIX04705
Salmo salar	Salmoniformes	Beta-defensin 3	QIX04706
Salmo salar	Salmoniformes	Beta-defensin 4	QIX04707
Salmo salar	Salmoniformes	Beta-defensin 5a	QIX04708
Oncorhynchus mykiss	Salmoniformes	Beta-defensin 1	CAK54950
Oncorhynchus mykiss	Salmoniformes	Beta-defensin 2	CAR82090
Oncorhynchus mykiss	Salmoniformes	Beta-defensin 3	CAR82091
Oncorhynchus mykiss	Salmoniformes	Beta-defensin 4	CAR82092
Takifugu rubripes	Tetraodontiformes	Beta-defensin-like 1	CAJ57646
Tetraodon nigroviridis	Tetraodontiformes	Beta-defensin-like 1	CAJ57644
Tetraodon nigroviridis	Tetraodontiformes	Beta-defensin-like 2	CAJ57645
Paralichthys olivaceus	Pleuronectiformes	Beta-defensin 1	ADA84138
Paralichthys olivaceus	Pleuronectiformes	Beta-defensin 3	ADA84140
Paralichthys olivaceus	Pleuronectiformes	Beta-defensin 4	ADA84141
Scophthalmus maximus	Pleuronectiformes	Beta-defensin-like 1	ENSSMAT00000025713.1
Scophthalmus maximus	Pleuronectiformes	Beta-defensin 2	MW648585
Ictalurus punctatus	Siluriformes	Beta-defensin	APU66342
Oplegnathus fasciatus	Centrarchiformes	Beta-defensin	AJA33388
Siniperca chuatsi	Centrarchiformes	Beta-defensin	ACO88907
Oryzias latipes	Beloniformes	Beta-defensin	ACG55699
Liza haematocheila	Mugiliformes	Beta-defensin	AIK66783
Acipenser dabryanus	Acipenseriformes	Beta-defensin	QBJ27760
Seriola lalandi	Carangiformes	Beta-defensin-like 1	ENSSLDT00000023067.1
Seriola lalandi	Carangiformes	Beta-defensin-like 2	ENSSLDT00000026660.1
Mus musculus	Rodentia	Beta-defensin 1	AAB72003
Mus musculus	Rodentia	Beta-defensin 4	NP_062702
Homo sapiens	Primates	Beta-defensin 1	AAB49758
Homo sapiens	Primates	Beta-defensin 2	AAC33549
Ovis aries	Artiodactyla	Beta-defensin 1	DAB41723

Gallus gallus	Galliformes	Beta-defensin 1	AAT48925
Bothrops jararaca	Squamata	Beta-defensin 2	AGF25387
Crassostrea gigas	Ostreida	Beta-defensin 1	AEE92768
Homo sapiens	Primates	Alpha-defensin 1	NP_004075
Mus musculus	Rodentia	Alpha-defensin 1	NP_034161
Papio anubis	Primates	Theta-defensin a	ACJ12913
