# Supplementary material for: Antimicrobial Peptides: Identification of two Beta-Defensins in a Teleost Fish, the European Sea Bass (Dicentrarchus labrax)
Source: Pharmaceuticals (Basel). 2021 Jun 14;14(6):566. doi: 10.3390/ph14060566 (PMC8231796; doi:10.3390/ph14060566)
Supplement: Supplementary file 1 [file pharmaceuticals-14-00566-s001.zip › Supplementary Table3.pdf]

Table S3 Accession numbers of genes used in the synteny analysis of beta-defensins 1 and 2.

	Species				
**Genes**	***D. labrax***	***L. calcarifer***	***S. aurata***	***S. lalandi*[b]**	***S. maximus*[b]**
defb1	ENSDLAG00005026052	ENSLCAG00010025743[a]	ENSSAUG00010014090	ENSSLDG00000017411[a]	ENSSMAG00000015557[a]
defb2	ENSDLAG00005001996	ENSLCAG00010006231	ENSSAUG00010026539	ENSSLDG00000020114	ENSSMAG00000013172
atp1b2b	ENSDLAG00005026068	ENSLCAG00010025763	ENSSAUG00010015305	ENSSLDG00000017566	ENSSMAG00000015277
CAPN6	ENSDLAG00005001788	ENSLCAG00010006146	ENSSAUG00010025836	ENSSLDG00000020262	ENSSMAG00000013231
chrdl2	ENSDLAG00005002011	ENSLCAG00010006252	ENSSAUG00010026542	ENSSLDG00000020064	ENSSMAG00000013155
cnksr2b	ENSDLAG00005025976	ENSLCAG00010025729	ENSSAUG00010014051	ENSSLDG00000017251	ENSSMAG00000015588
il13ra2	ENSDLAG00005001759	ENSLCAG00010006126	ENSSAUG00010025832	ENSSLDG00000020312	ENSSMAG00000013307
KCNAB3	ENSDLAG00005002038	ENSLCAG00010006338	ENSSAUG00010026552	ENSSLDG00000020006	ENSSMAG00000013080
lrch2	ENSDLAG00005001681	ENSLCAG00010006112	ENSSAUG00010025830	ENSSLDG00000020331	ENSSMAG00000013376
mepce	ENSDLAG00005001988	ENSLCAG00010006223	ENSSAUG00010026538	ENSSLDG00000020120	ENSSMAG00000013182
mogat3b	ENSDLAG00005026062	ENSLCAG00010025759	ENSSAUG00010015302	ENSSLDG00000017526	ENSSMAG00000015327
neu3.1	ENSDLAG00005002025	ENSLCAG00010006274	ENSSAUG00010026547	ENSSLDG00000020043	ENSSMAG00000013071
nyap1	ENSDLAG00005026065[a]	ENSLCAG00010025761[a]	ENSSAUG00010015303	ENSSLDG00000017545	ENSSMAG00000015310[a]
or121	ENSDLAG00005026051	ENSLCAG00010025739	ENSSAUG00010014085[a]	ENSSLDG00000017386[a]	ENSSMAG00000015567[a]
PAK3	ENSDLAG00005001894	ENSLCAG00010006195	ENSSAUG00010025886	ENSSLDG00000020143	ENSSMAG00000013188
rnf121	ENSDLAG00005025916	ENSLCAG00010025715	ENSSAUG00010014027	ENSSLDG00000017174	ENSSMAG00000015786
rnf167	ENSDLAG00005026054	ENSLCAG00010025746	ENSSAUG00010014093	ENSSLDG00000017455	ENSSMAG00000015502
rrm1	ENSDLAG00005025894	ENSLCAG00010025699	ENSSAUG00010013817	ENSSLDG00000017093	ENSSMAG00000015872
spag7	ENSDLAG00005002002	ENSLCAG00010006239	ENSSAUG00010026541	ENSSLDG00000020103	ENSSMAG00000013168
srrt	ENSDLAG00005026057	ENSLCAG00010025754	ENSSAUG00010014122	ENSSLDG00000017477	ENSSMAG00000015342
trpc2b	ENSDLAG00005025880	ENSLCAG00010025683	ENSSAUG00010013753	ENSSLDG00000017035	ENSSMAG00000015927
xrra1	ENSDLAG00005002022	ENSLCAG00010006262	ENSSAUG00010026544	ENSSLDG00000020051[c]	ENSSMAG00000013136

defb: beta-defensin; *atp1b2b*: ATPase Na+/K+ transporting subunit beta 2b/ Sodium/ potassium-transporting ATPase subunit beta-2; *CAPN6*: Calpain 6; *chrdl2*: chordin 2; *cnksr2b*: Connector enhancer of kinase suppressor of Ras 2b; *il13ra2*: Interleukin 13 receptor, alpha 2; *KCNAB3*: Potassium voltage-gated channel subfamily A regulatory beta subunit 3; *lrch2*: Leucine-rich repeats and calponin homology (CH) domain containing 2; *mepce*: Methylphosphate capping enzyme/ 7SK snRNA methylphosphate capping enzyme; *mogat3b*: Monoacylglycerol O-acyltransferase 3b/ Diacylglycerol O-acyltransferase 2; *neu3.1*: Sialidase 3 (membrane sialidase), tandem duplicate 1/ Neuraminidase 3; *nyap1*: Neuronal Tyrosine Phosphorylated Phosphoinositide-3-Kinase Adaptor 1; *or121*: Odorant receptor, family E, subfamily 121, member 1; *PAK3*: p21 (RAC1) activated kinase 3/ Serine/threonine-protein kinase PAK 3; *rnf121*: Ring finger protein 121; *rnf167*: Ring finger protein 167/ E3 ubiquitin-protein ligase RNF167; *rrm1*: Ribonucleotide reductase M1 polypeptide/ Ribonucleoside-diphosphate reductase large subunit; *spag7*: Sperm associated antigen 7; *srrt*: Serrate RNA effector molecule homolog; *trpc2b*: Transient receptor potential cation channel subfamily C member 2b/ Short transient receptor potential channel 2-like; *xrra1*: X-ray radiation resistance associated 1. [a] Predicted gene; [b] Reverse strand; [c] Incomplete gene.
